# The Role of Genetically Modified Mesenchymal Stem Cells in Urinary Bladder Regeneration

**DOI:** 10.1371/journal.pone.0138643

**Published:** 2015-09-23

**Authors:** Devon C. Snow-Lisy, Edward C. Diaz, Matthew I. Bury, Natalie J. Fuller, Jessica H. Hannick, Nida Ahmad, Arun K. Sharma

**Affiliations:** 1 Ann & Robert H. Lurie Children's Hospital of Chicago, Division of Pediatric Urology, Chicago, IL, United States of America; 2 Department of Urology, Loyola University Health System, Maywood, IL, United States of America; 3 Northwestern University Feinberg School of Medicine, Department of Urology, Chicago, IL, United States of America; 4 Northwestern University, Simpson Querrey Institute for BioNanotechnology, Chicago, IL, United States of America; 5 Northwestern University, Department of Biomedical Engineering, Evanston, IL, United States of America; National Cancer Institute, UNITED STATES

## Abstract

Recent studies have demonstrated that mesenchymal stem cells (MSCs) combined with CD34^+^ hematopoietic/stem progenitor cells (HSPCs) can function as surrogate urinary bladder cells to synergistically promote multi-faceted bladder tissue regeneration. However, the molecular pathways governing these events are unknown. The pleiotropic effects of Wnt5a and Cyr61 are known to affect aspects of hematopoiesis, angiogenesis, and muscle and nerve regeneration. Within this study, the effects of Cyr61 and Wnt5a on bladder tissue regeneration were evaluated by grafting scaffolds containing modified human bone marrow derived MSCs. These cell lines were engineered to independently over-express Wnt5a or Cyr61, or to exhibit reduced expression of Cyr61 within the context of a nude rat bladder augmentation model. At 4 weeks post-surgery, data demonstrated increased vessel number (~250 vs ~109 vessels/mm^2^) and bladder smooth muscle content (~42% vs ~36%) in Cyr61OX (over-expressing) vs Cyr61KD (knock-down) groups. Muscle content decreased to ~25% at 10 weeks in Cyr61KD groups. Wnt5aOX resulted in high numbers of vessels and muscle content (~206 vessels/mm^2^ and ~51%, respectively) at 4 weeks. Over-expressing cell constructs resulted in peripheral nerve regeneration while Cyr61KD animals were devoid of peripheral nerve regeneration at 4 weeks. At 10 weeks post-grafting, peripheral nerve regeneration was at a minimal level for both Cyr61OX and Wnt5aOX cell lines. Blood vessel and bladder functionality were evident at both time-points in all animals. Results from this study indicate that MSC-based Cyr61OX and Wnt5aOX cell lines play pivotal roles with regards to increasing the levels of functional vasculature, influencing muscle regeneration, and the regeneration of peripheral nerves in a model of bladder augmentation. Wnt5aOX constructs closely approximated the outcomes previously observed with the co-transplantation of MSCs with CD34^+^ HSPCs and may be specifically targeted as an alternate means to achieve functional bladder regeneration.

## Introduction

Neurogenic urinary bladder, radiation or interstitial cystitis, severe incontinence, and urinary bladder cancer provide the impetus for urinary bladder regeneration strategies.[1–2] For those patients that are refractory to conservative therapy, the current standard of care is for bladder replacement or augmentation, depending upon the underlying pathology. These invasive surgical procedures utilize a portion of bowel to either replace or augment the existing bladder. Unfortunately, the use of bowel is fraught with numerous short and long-term complications, including metabolic derangements, infection, stone formation, small bowel obstruction, perforation, and an increased incidence of aggressive tumor development.[3,4] These obstacles have provided the motivation to investigate alternative approaches including cutting-edge tissue engineering strategies to create functional bladder tissue.

A clinical trial utilizing autologous sources of bladder cells obtained from spina bifida patients were expanded ex vivo and then combined with synthetic scaffolds to provide the first clinical foray into this field.[5] Although novel in approach, the outcomes of the study were inconclusive. A second iteration of this study was recently conducted in a phase II multi-center trial. Autologous bladder cells from spina bifida patients were again seeded onto synthetic scaffolds and implanted in spina bifida patients.[6] Unfortunately there were no statistically significant improvements in physiological bladder parameters including compliance and capacity at 12 or 36 months post-grafting. Of great clinical importance, adverse events occurred in all patients, including bowel obstruction in 40% of these children. These poor outcomes have led to the re-evaluation of the choice of cell types to utilize in this setting as well as the type of scaffold that would provide the greatest clinical benefit. This has also provided an interest in the mechanisms behind optimal tissue regeneration in the bladder.[7–10]

The plastic nature of mesenchymal stem cells (MSCs) has demonstrated promising results across a variety of clinical areas including bladder augmentation, heart failure or attack, ischemic stroke, urinary incontinence and even mediating kidney transplant rejection.[11–15] While initially thought to impact tissue regeneration by cellular engraftment and differentiation, it now appears that the regenerative and therapeutic effects of MSCs may primarily be due to paracrine-based mechanisms.[7, 16–20] The MSC secretome and its effect on angiogenesis, peripheral nerve regeneration, collagen deposition, and immunomodulation have not been completely characterized. We have previously shown that co-transplantation of MSCs with bone marrow derived CD34^+^ hematopoietic stem/progenitor cells (HSPCs) enhances tissue vascularization, urothelium regeneration, bladder smooth muscle regeneration, and peripheral nerve regeneration in a bladder augmentation model at the gross level.[7] However, the signaling pathways involved in these parameters of tissue regeneration have not been delineated including those that directly affect angiogenesis and overall tissue development. Hence, we focused efforts to evaluate whether the pro-angiogenic effects of Cysteine-rich angiogenic inducer 61 (Cyr61) and the pleiotropic functions of Wnt5a are potentially important factors involved in tissue regeneration following bladder augmentation.

Cyr61, also known as CCN1, is a secreted extracellular matrix protein regulating numerous elements important for wound healing and tissue regeneration. Depending on its interaction with cell and environment specific integrins and heparin sulfate proteoglycans, Cyr61 has been shown to promote cell adhesion, survival, proliferation, senescence, angiogenesis, and apoptosis.[21–24] Cyr61 decreases fibrosis during cutaneous wound healing via induction of fibroblast senescence through binding to integrin α_6_β_1_.[25] Cyr61 is most well-known for its role in angiogenesis.[20, 25–27] Dissection of the murine bone marrow derived MSC secretome has lead to the identification of Cry61 as a main component of the angiogenic response.[20] In tonsil derived MSCs, Cyr61 was shown to induce endothelial cell migration and tube formation for angiogenesis via integrin α_v_β_3_ and AMPK.[22] Interestingly, Cyr61 expression is increased after mechanical stretching of bladder smooth muscle cells, leading to increases in vascular endothelial growth factor (VEGF; a potent angiogenic growth factor), integrin α_v_, and α-smooth muscle actin.[23] Furthermore, Cyr61 recruits CD34^+^ HSPCs, which may be an additional method by which it enhances angiogenesis.[28] Cyr61 is likely an important modulator in bladder regeneration due to its actions within angiogenic blood vessels and neighboring MSCs following augmentation cystoplasty.[29]

The Wnt family of proteins encodes highly hydrophobic, lipid modified, secreted glycoproteins that are capable of diverse developmental and maintenance processes throughout the lifespan of metazoan organisms. [30–33] Dysfunctional expression of Wnt genes is the basis of some cancers including mammary tumors, improper neural development, diabetes, and other degenerative disorders.[34–37] These pleiotropic effects of Wnt genes have been examined at the molecular level in a variety of systems. The putative binding partners for Wnt ligands are the frizzled (Fzd) family of receptors. The ligand-receptor interactions have been partially delineated for Wnt and Fzd proteins within a variety of cell and tissue systems. This includes stem cells within the hematopoietic stem cell niche as well as more differentiated cell types associated with angiogenesis.[38–42] We have previously demonstrated the cloning of human Wnt5a from the stromal cells that line the bone marrow cavity.[38] The constitutive expression of Wnt5a caused an approximate 30 fold increase in mixed lineage hematopoietic progenitors than were initially derived from primitive bone marrow CD34^+^ HSPCs within an in vitro co-culture system. We went onto to further demonstrate that the primitive CD34^+^ HSPCs themselves also express Wnt5a and may engage in paracrine signaling within the local bone marrow environment. More recently, it has been demonstrated that Wnt5a plays a role in angiogenesis. In the study by Masckauchan et al, data demonstrate that Wnt5a is expressed by human primary endothelial cells and in murine vasculature.[40] The expression of exogenous Wnt5a in human endothelial cells caused the cells to undergo angiogenesis via the non-canonical Wnt signaling pathway. The expression of Wnt5a enhanced capillary network formation while a reduction in Wnt5a decreased capillary network formation and reduced endothelial cell migration. Wnt5a has also been shown to up-regulate the expression of pro-angiogenic modulators including the Tie-2 receptor, matrix metalloproteinase-1 and interstitial collagenase. A second study demonstrated that Wnt5a promotes the proliferation and survival of endothelial cells. Ishikawa et al describe the lack of viability in mice that did not express Fzd5, one of the receptors for Wnt5a, due to defects in yolk sac angiogenesis.[42] Wnt5a can also act through the Fzd4 receptor as well, as this becomes relevant to our studies in bladder.[43]

Hence, we wish to independently examine the mechanistic roles of Cyr61 and Wnt5a in genetically modified MSCs within the context of a nude rat bladder augmentation model. We will specifically examine the impact of modified Cyr61 and Wnt5a expression with regards to bladder physiology, bladder smooth muscle content, peripheral nerve, urothelial regeneration, and tissue angiogenesis in regenerating bladder tissue. Finally, we would like to ascertain whether MSCs over-expressing Wnt5a can mimic the effects previously demonstrated utilizing MSC/CD34^+^ HSPCs seeded scaffolds within the aforementioned augmentation model.

## Materials and Methods

### Ethics Statement

All animal procedures were performed as per protocols approved by the Ann & Robert H. Lurie Children’s Hospital Research Center Institutional Animal Care and Use Committee (IACUC # 13–046.0).

### Cyr61 and Wnt5a Over-expression Cell Lines

The cDNA encoding homo sapiens Cyr61 (Genbank Accession # BC001271.1) was isolated by PCR amplification from Human Placenta QUICK-Clone cDNA (Clontech Laboratories, CA, USA). The PCR amplification profile consisted of the following: [94°C-45 sec]-1 cycle; [94°C-45 sec; 60°C-45 sec; 72°C-2 min]-30 cycles; 72°C-10 min; 4°C-hold utilizing the PfuUltra High Fidelity DNA Polymerase (Agilent Technologies, CA, USA). Primers used for amplification were: Cyr61for1 5’-CTCCAGAATTCATGAGCTCCCGCATCGCCA-3’ Cyr61rev1 5’-GCTCCAGGATCCTTAGTCCCTAAATTTGTGAATGTCAT-3.’ Isolated amplicons were gel purified (Qiaquick Gel Purification Kit; Qiagen Inc., CA, USA) and enzymatically digested with BamH1 and EcoR1 (New England Biolabs Inc., MA, USA) for ligation into the pre-digested pCDH-EF1-MCS-IRES-GFP cDNA Cloning and Expression HIV lentiviral vector [System Biosciences Incorporated (SBI), CA, USA].

Homo sapiens Wnt5a (Genbank Accession # BC064694.1) was isolated by Swa1 and Not1 (New England Biolabs Inc.) digestion of the GC-B0116 plasmid (GeneCopoeia, MD, USA). The Wnt5a digestion product was gel purified and ligated into the previously described, pre-digested lentiviral vector using Swa1 and Not1 restriction enzymes. Cyr61 and Wnt5a gene sequences were verified through DNA sequencing performed at the Northwestern University Genomics Core Facility. Over-expression plasmids for Cyr61 and Wnt5a are termed as pOX-Cyr61, and pOX-Wnt5a, respectively.

### Cyr61 Knockdown Cell Line

Three knockdown plasmids were created for Cyr61, and are referred to as pKD-Cyr61a,-b and-c. The cDNA sequence for Cyr61 was scanned for amino acid dinucleotides and 21 downstream base pairs (bp) were used to create a sense oligonucleotide. A 12 bp spacer (CTTCCTGTCAGA) was used for the loop and the antisense sequence for 21 bp was added after the space and a tail of five thymines was added to the 3’ end for a termination signal. Overhangs were designed to facilitate ligation of annealed oligos into BamHI and EcoRI cloning sites on pGreenPuro shRNA HIV Lentivector (SBI). The authenticity of DNA sequences was verified through the Northwestern University Genomics Core Facility.

### Viral Supernatant Production

For viral supernatant production, 293TN producer cells (SBI) were seeded on 100 mm plates in Opti-MEM (Invitrogen) media and allowed to reach ~80–90% confluence within 24 hours. After achieving 80–90% confluence, the plates were transfected utilizing Lipofectamine (LifeTechnologies, Inc.) according to manufacturer’s instructions. Transfection utilized a mixture of lentiviral packaging vectors and **target vector(s)** (pOX-Cyr61, pOX-Wnt5a, or equal mixture of pKD-Cyr61a,-b, and-c). Plasmid molar ratio (target vector(s)/MDG/CMV) 1.5/0.5/2.0 was utilized. Cells were incubated and GFP expression was noted to be >80% for all transfectants utilizing fluorescent microscopy after 24 hours. Viral supernatant was collected at 24 and 48 hours post-transfection and filtered through a 0.45μm Acrodisc syringe filter (Sigma-Aldrich, MO, USA).

### Viral Transduction of MSCs

12 well plates were coated with 10 μg/cm^2^ retronectin (Clontech Laboratories). Viral supernatant was then bound to the retronectin plate via spinoculation at 1500g x 2 hours at 32°C with 1ml per well. The supernatant was removed and the plate washed with phosphate buffered saline (PBS) and 2% BSA. BM-MSCs (<p8; Lonza, MD, USA) were seeded at a density of 1x10^4^ cells/cm^2^. Plates were incubated and monitored with fluorescence microscopy for 48 hours for GFP expression. Following expansion with MSC Growth Media (Lonza) cells were sorted for GFP using FACS through the Robert H. Lurie Comprehensive Cancer Flow Cytometry Facility (Northwestern University). Unmanipulated MSCs were cultured in the aforementioned MSC Growth Media.

### Assessment of Gene Transfer

Cell lysates containing the aforementioned lentiviral cell lines were prepared for Western blot analysis by resuspending 2x10^7^ scraped MSCs in ice cold Radio-Immunoprecipitation Assay (RIPA) buffer (1ml) as per manufacturers protocol (Santa Cruz Biotechnology (SCBT), CA, USA). Protein content was then analyzed per manufacturer’s protocol with a BCA Protein Assay Kit (LifeTechnologies, Inc.).

A reduced Western blot was performed utilizing the NuPAGE SDS-PAGE System (Invitrogen). Samples (30μg of total protein) were then loaded into a pre-cast 10% NuPAGE Bis-Tris Gel with MOPS running buffer and NuPage antioxidant (Invitrogen). Gel transfer was performed utilizing the iBlot system (Invitrogen). A PVDF membrane (Invitrogen) was used at 20V with a run time of 7 minutes. The blots were then blocked with 5% nonfat milk in Tris-Buffered Saline (TBS) with 0.2% Tween (TBST) at 4°C for 1 hour. After rinsing with TBST the blots were incubated with primary antibody overnight at 4°C. Cyr61 primary antibody (SCBT) was diluted 1:400 in 5% milk in TBS. Wnt5a primary antibody (Abcam, MA, USA) was diluted 1:400 in 1% milk in TBS. After blot washes with TBST, the secondary antibody was incubated with the blots for 1 hour at room temperature. Secondary antibody (Abcam) was diluted 1:2000 in 1% milk in TBS for the Wnt5a blot, and diluted 1:3000 in 5% milk in TBS for the Cyr61 blot. Detection was then performed per manufacturer protocol using ECL western blotting detection reagents and analysis systems (GE Healthcare Life Sciences, PA, USA). CL-XPosure films (LifeTechnologies) were exposed for 15 and 30 seconds and 1, 3, and 5 minutes.

The levels of the housekeeping protein β-tubulin were determined by stripping the blots with Restore Western Blot Stripping Buffer (LifeTechnologies) for 5–7 minutes. The blots were then blocked for 2 hours at 4°C with 5% nonfat milk and TBST. The primary antibody β-tubulin (Abcam) was incubated overnight at a dilution of 1:500 at 4°C on an orbital shaker. The blots were then rinsed and incubated with the IgG-HRP secondary antibody (Abcam) at a dilution of 1:2000 in 1% milk in TBS for 1 hour at room temperature. After washing, detection was performed as previously described.

### POC Scaffold Synthesis and MSC Seeding

Poly (1,8-octanediol-cocitrate) (POC) was synthesized as previously reported in detail.[7]. Polymerized POC was cut into 0.50cm x 0.50cm x 0.2cm (length x width x thickness) scaffolds and unreacted monomers were leached by incubation with DMEM media (Lonza) which was changed every 6 hours over a 24 hour period. The scaffolds were seeded with 1.5x10^4^ MSCs/cm^2^ and allowed to grow in MSC media for 7–8 days prior to augmentation cystoplasty. If scaffolds had poor cell growth as seen by light microscopy, re-seeding was performed as needed to achieve confluence prior to bladder grafting.

### Nude Rat Bladder Augmentation Model

8–10 week old athymic female rats (n = 5 per group, Charles River Laboratories International, MI, USA) underwent bladder augmentation as previously described.[7] Briefly, following box induction with inhaled isoflurane anesthesia (2–3%) animals were placed on a heating pad and maintained with nose-cone isoflurane anesthesia (1–3%). A 1.5-cm lower midline abdominal incision was made. Pre-augmentation urodynamic testing and capillaroscopy was performed. The anterior incision of a 50–60% supratrigonal cystectomy was made and capillaroscopy was performed at the dome. Following this procedure, the remaining dome of the bladder was excised. The bladder was then augmented with the previously described MSC seeded POC scaffold using 7–0 polydioxanone (PDS II; Ethicon, NJ, USA) suture in a running watertight fashion. Perivesicle fat was used to cover the graft and adjacent bladder. The abdominal wall and skin were closed with interrupted 4–0 chromic suture (Ethicon). Buprenorphine (1mg/kg) was administered subcutaneously to all animals. After 4 or 10 weeks, animals underwent repeat anesthesia, urodynamic testing, and capillaroscopy followed by euthanasia via cervical dislocation. The bladder was harvested for further analyses. The different numbers of animals for analysis were solely due to animal mortalities. Animals were sacrificed by CO_2_ overdose followed by surgical dislocation to ensure humane termination. An n = 5 animals for all groups were used for statistical analysis.

### Functional Evaluation of Bladders with Urodynamics

Urodynamic testing was performed as previously described.[44] After exposure of the bladder, a 27 gauge needle was placed intravesically through the dome. The needle was then connected to an Elite Syringe Pump (Harvard Apparatus, MA, USA), a physiological pressure transducer (SP844, MEMSCAP, NC, USA), and a bridge amplifier (Model FE221; AD Instruments, Dunedin, New Zealand). Continuous readings of the intravesical pressure were obtained with LabChart 7.3 Software (AD Instruments). The fill rate for each study ranged from 150–200 μL/min.

### Capillariscopy

Qualitative evaluation of the bladder microvasculature was obtained with a CapiScope HVCS handheld video capillaroscopy system (KK Technology, United Kingdom). The CapiScope was used to obtain videos of the native bladder microvasculature of the dome. Prior to sacrifice, regenerated bladders were also evaluated. A vertical incision was made through the anterior bladder wall exposing native and regenerated bladder tissue. The regenerated tissue, as identified by remaining graft, was then imaged with the CapiScope.

### Histological Analysis of Augmented Bladders

All harvested organs were immediately fixed in 10% buffered formalin phosphate (Fisher Scientific, MA, USA). The samples were then dehydrated through graded ethanol exchanges followed by paraffin embedding as previously described.[45] Tissues were then sectioned (10μm) using a RM2125 RT microtome (Leica Biosystems, IL, USA). The samples were then treated with xylenes, graded ethanol washes and deionized water. Sections were stained with Masson’s trichrome (Sigma-Aldrich) or via immunofluorescence staining with anti-neuronal antibody βIII tubulin (Covance Inc., NJ, USA) as previously described.[7]

For Masson’s trichrome, the samples were placed in Bouin’s solution for 15 min, rinsed under running water, placed in hematoxylin for 5 min, rinsed with water, stained with Scarlet-Acid Fuchsin for 5 min, rinsed with deionized water then placed in a mixture of phosphotungstic acid/phosphomolybdic acid, followed by Analine Blue staining. The samples were then finally washed with 1% acetic acid, rinsed with 95–100% ethanol and rinsed in xylene. A coverslip was then applied and secured with 2–3 drops of Permaslip (Alban Scientific Inc., MO, USA).

For immunofluorescence staining, sections were blocked for 15 min with bovine serum albumin (5 mg/mL) followed by a 40 min incubation at room temperature with βIII tubulin (Covance Inc.), the primary antibody, at a dilution of 1:2000. After washing with Dulbecco's Phosphate-Buffered Saline (DPBS), slides were incubated for 30 minutes with goat anti-rabbit Alexa Fluor 488 (Invitrogen), the secondary antibody, at a dilution of 1:400. Following this the slides were rinsed with DPBS, air-dried and mounted with Vectashield (Vector Laboratories, CA, USA).

### Quantitative Evaluation of Augmented Bladders

Full thickness bladder tissue specimens were evaluated as previously described.[7] Images were obtained by scanning through both the native and regenerated tissue with a Nikon Eclipse 50i microscope (Nikon Instruments, NY, USA) and Spot Advanced Imaging software (Diagnostic Instruments, MI, USA). Brightfield microscopic images were 1,600 x 1,200 pixels, a bit depth of 24, and were quantified with Adobe Photoshop CC (Adobe Systems Inc., CA, USA). Immunofluorescent microscopic images were 1,600 x 2,000 pixels, a bit depth of 24, and were quantified with Spot Advanced Imaging software (Diagnostic Instruments).

Vascular quantification was achieved by analyzing the Masson’s trichrome images. Vessel numbers were outlined using the pen tool (Adobe Photoshop) in ten random images each of the native and regenerated bladder tissue. Individual vessels were selected and quantified with the image histogram tool to acquire pixel count per vessel. Data are represented as mean number of vessels per square millimeter and mean percent vasculature (means ± SEM).

Bladder smooth muscle/collagen quantification was achieved by analyzing the Masson’s trichrome images. In Adobe Photoshop, the contrast of red to blue pixels was enhanced by a two fold elevation of magenta levels followed by two fold depression of cyan levels in the red/magenta spectra and the reverse in the blue/cyan spectra. Prior to evaluation of the muscle/collagen ratio, the images were edited to remove urothelial cells, red blood cells, debris and vasculature. The color-range selection tool (fuzziness level 115%) was then used to select the red or blue pixels of the entire image. These pixels were quantified using the image histogram tool and the percent muscle and density values were calculated from these data. Data are expressed as means ±SEM for percent muscle (red pixel count/red+blue pixel count) and a density score. The density score describes the density of the tissue (i.e. how tightly the muscle and collagen are packed) and normalizes this against control bladder tissue (mean, n = 3 control samples). Density score is defined as [(sample red + blue pixel count)/(mean normal red+blue pixel count)] such that control tissue has a density score of 1.

Neural quantification was achieved using the immunofluorescent βIII tubulin stained images. The border between native and regenerated bladder tissue was identified and the entire regenerated tissue was scanned for peripheral nerve tissue that were at least 3 cells long and images were obtained when they were present. Peripheral nerves were counted and measured using Spot Advanced Imaging software. Maximum peripheral nerve regeneration distance was quantified by measuring the shortest distance between the native-regenerated tissue border and the farthest instance of βIII tubulin^+^ staining for each animal. Data are expressed as means ±SEM for instances of βIII tubulin^+^ staining for nerve length, and maximum nerve regeneration distance.

Urothelium quantification was achieved using the Masson’s trichrome images. Three 10x images were obtained spanning the length of the regenerated bladder tissue. Ten measurements per image were obtained by randomly spanning the length of regenerated bladder tissue. The distance from the urothelial lumen to the superficial aspect of the lamina propria was measured for each image using Spot Advanced Imaging software. Data are expressed as means ±SEM.

Functional quantification was achieved by analyzing urodynamics tracings in LabChart 7.3. Bladder capacity was measured by calculating the volume infused prior to first urine leak as described previously.[46] Peak voiding pressures were measured as the highest pressure at time of void. A measure of compliance was calculated as the percentage of bladder volume that was infused at pressures ≤ 20 cmH_2_O.[47–49] Due to the highly invasive nature of this functional bladder testing, urodynamics were obtained pre-operatively and immediately prior to euthanasia while the animals were under isoflurane anesthesia.

### Statistical Analysis

Differences between groups were determined using Student’s *t* test or ANOVA with the Tukey-Kramer post-hoc test. P values less than 0.05 were considered statistically significant. Analyses were performed with SAS 9.4 software (SAS Institute).

## Results

### Western Blot Analyses

**Fig 1:**
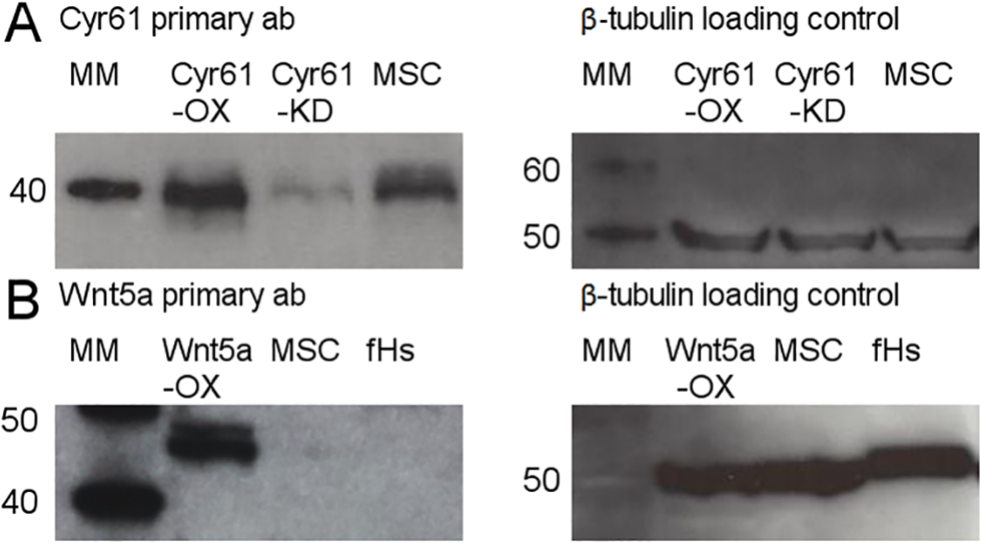
MSC construct validation. (A) Western blot analysis of the Cyr61OX MSC construct demonstrates significant over-expression of Cyr61 as compared to unmanipulated MSCs (expected molecular weight of ~40kDa). The Cyr61KD MSC construct demonstrates significantly reduced expression of Cyr61. β-tubulin loading control confirmed equivalent protein loading amongst samples. Unmanipulated MSCs were cultured in MSC Growth Media (Lonza). (B) Western blot analysis of the Wnt5aOX MSC construct demonstrates significant over-expression as compared to unmanipulated MSCs (expected molecular weight of 45kDa). The minimal expression of Wnt5a in unmanipulated MSCs is readily apparent upon longer exposure times. Protein lysate from the fibroblast cell line fHs 173We was used as a negative control compared to Wnt5a constructs. β-tubulin loading control confirmed equivalent protein loading amongst samples.

Western blot data demonstrated the over-expression and knockdown the of Cyr61 protein expression in the Cyr61OX/MSC and Cyr61KD/MSC cell lines (Fig 1A). An approximate 40kDa protein band confirming the expression of Cyr61 was visible upon exposure. The Wnt5aOX/MSC cell line demonstrated robust over-expression compared to unmanipulated MSCs with strong expression at the expected 45kDa molecular weight level (Fig 1B). β-tubulin loading controls further demonstrated that equivalent amounts of protein were loaded amongst all samples, visible at the 50kDa molecular weight level. Detection of Wnt5a in unmanipulated MSCs was noticeable upon longer exposure of films. There were several attempts to create a Wnt5aKD/MSC cell line however Wnt5aKD transduced MSCs failed to thrive in culture several days following transduction. Hence, this cell line was not included within this study.

### Blood Vessel Characterization

**Fig 2:**
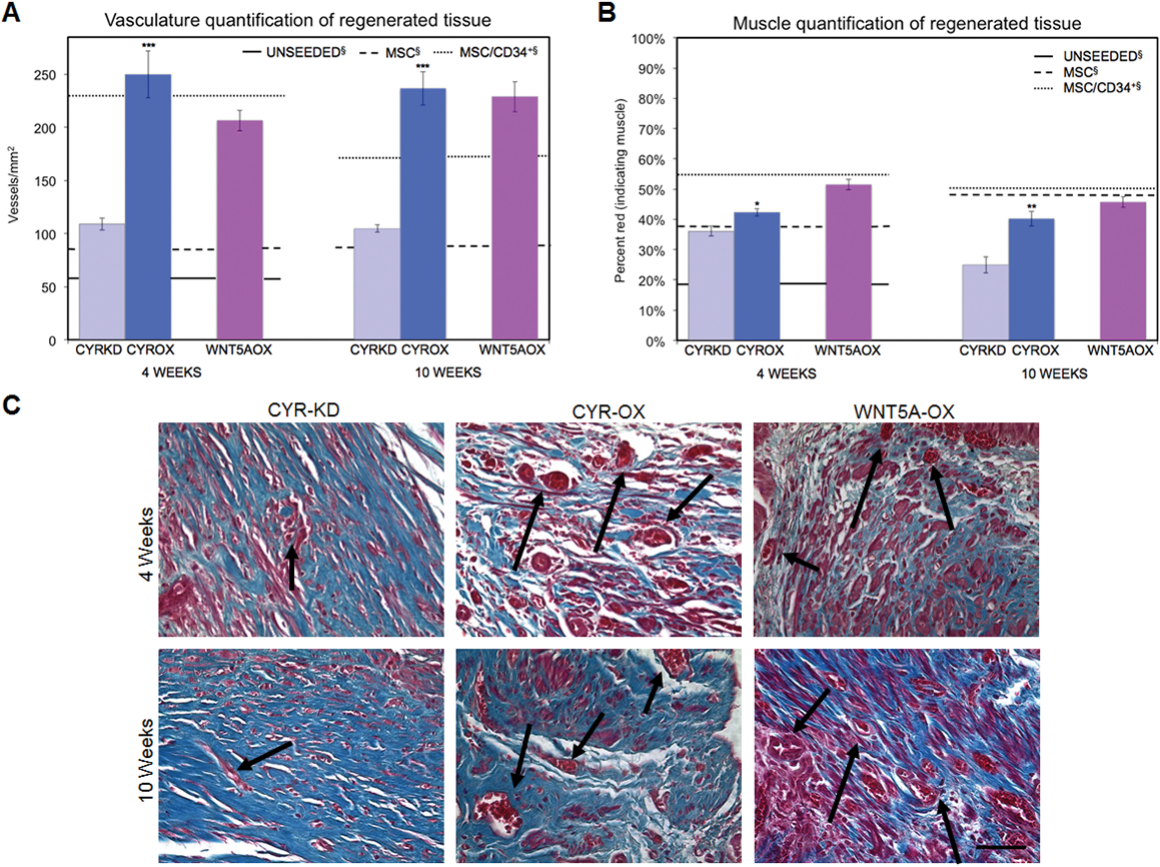
Effects of Cyr61 and Wnt5a on regenerating vasculature and musculature. (A) Cyr61OX and Wnt5aOX showed early and persistent increased vasculature, comparably greater than unmanipulated MSCs, with numbers of vessels/mm^2^ similar to MSC/CD34^+^ HSPC grafts at 4 weeks. (§ Unseeded, MSC and MSC/CD34^+^ HSPC data, shown as solid and dotted lines, previously reported [7]). At both 4 and 10 weeks, Cyr61KD demonstrated significantly decreased vasculature as compared to Cyr61OX. Data shown as means ± SEM; ***P<0.001 for Cyr61KD vs Cyr61OX. (B) Wnt5aOX demonstrated mean muscle content most comparable to MSC/CD34^+^ HSPC grafts [percent red (muscle): (red (muscle) pixels / red (muscle) + blue (collagen) pixels)]. (§ Unseeded, MSC and MSC/CD34^+^ HSPC data, shown as solid and dotted lines, previously reported [7]). Cyr61KD demonstrated a significant decrease in muscle percentage from 4 to 10 weeks (p<0.05), with levels significantly lower than Cyr61OX at both time points. Data shown as means ± SEM; *P<0.05 and **P<0.01 for Cyr61KD vs Cyr61OX. (C) Sample photomicrographs of Masson’s trichrome staining demonstrate differences in muscle/collagen content and level of vasculature (marked with black arrows) between Cyr61KD and the Cyr61OX and Wnt5aOX groups. Density score was greatest for Wnt5aOX at 4 weeks. Scale bar, 50 μm. (Unseeded, MSC and MSC/CD34^+^ HSPC images shown in S1 Fig).

Both increasing and decreasing Cyr61 expression resulted in greater levels of graft vasculature than previously observed with unmanipulated MSCs [7] in which increasing Cyr61 expression produced a much stronger effect. At 4 weeks, Cyr61KD vessel number and percent vasculature were 1.3x and 1.5x higher than MSC (108.8±5.5 vs 83.4±15.8 vessels/mm^2^ and 2.25±0.27% vs 1.46±0.16%) while Cyr61OX vessel number and percent vasculature were 3x and 4x higher than MSC (249.9±22.3 vs 83.4±15.8 vessels/mm^2^ and 5.78±0.29% vs 1.46±0.16%) (Fig 2A). For unmanipulated MSCs and Cyr61KD, there was no observed effect of graft duration (4 vs 10 weeks) on the level of vascularization. At 10 weeks, the number of vessels/mm^2^ remained stable for Cyr61OX but percentage vasculature increased (8.49±0.62% vs 5.78±0.24%, p<0.05), reflecting a shift towards larger vessels. Cyr61OX vessel number and percent vasculature were significantly greater than Cyr61KD at both time-points (p<0.001; p<0.001).

Wnt5aOX resulted in persistently high levels of vasculature in regenerated tissue which was comparatively greater than MSCs. At 4 weeks, vessel number and percent vasculature were 2.5x and 3.3x higher than MSC (206.4±9.7 vs 83.4±15.8 vessels/mm^2^ and 4.79±0.51% vs 1.46±0.16%) (Fig 2A). No significant increase was detected from 4 to 10 weeks.

Cyr61KD, Cyr61OX and Wnt5aOX all produced levels of vascularization greater than those previously reported for unmanipulated MSCs. In earlier findings, unseeded POC resulted in fewer vessels/mm^2^ than MSC-seeded POC (63.8±5.4 vs 83.4±15.8 vessels/mm^2^) and the addition of CD34^+^ HSPCs to MSC grafts increased graft vascularization [7] (S1 Fig). At 4 weeks, MSC/CD34^+^ HSPCs vessels/mm^2^ and percent vasculature were 2.8x and 5.5x greater than MSCs (230.9±11.4 vs 83.4±15.8 vessels/mm^2^ and 8.00±0.57% vs 1.46±0.16%). Cyr61OX and Wnt5aOX grafts showed numbers of vessels/mm^2^ similar to MSC/CD34^+^ HSPC grafts at this time-point, but with lower percent vasculature (5.78±0.29% and 4.79±0.51% vs 8.00±0.57%). This highlights the difference in time course observed for vascularization levels in MSC/CD34^+^ HSPC grafts versus the current vascularization-promoting cell lines. MSC/CD34^+^ HSPC grafts showed peak levels at the early time-point, with reduced vessels/mm^2^ and percent vasculature by 10 weeks (230.9±11.4 vs 178.3±10.7vessels/mm^2^ and 8.00±0.57% vs 5.31±0.44%). The current cell lines showed no reduction from 4 to 10 weeks; number of vessels/mm^2^ remained stable and percent vasculature either remained stable or, in the case of Cyr61OX, increased significantly.

Native tissue adjacent to the grafts showed similar vasculature characteristics across all groups at both the 4 and 10 week time-points. Capillaroscopy demonstrated functional vasculature in all groups at both 4 and 10 weeks. A sample video demonstrating capillaroscopy usage is presented in the Supporting Information (S1 File).

### Bladder Smooth Muscle Characterization

Modulating Cyr61 expression produced grafts with muscle content similar to unmanipulated MSC levels at 4 weeks, but comparatively lower by 10 weeks, either failing to reach >45% mean muscle content (Cyr61OX) or declining significantly to a mean value of only 25% (Cyr61KD). Previously, mean muscle content for MSC graft tissue increased from 4 to 10 weeks (38.4±1.0% at 4W vs. 47.2±0.6% at 10W) [7]; this trend was not observed with the Cyr61 constructs. Cyr61OX maintained muscle content (42.3±1.3% at 4W vs. 40.2±2.4% at 10W); consequently by 10 weeks mean muscle content remained <45%. In contrast, limiting the expression of Cyr61 resulted in a significant decrease from 4 to 10 weeks (36.1±1.6% vs. 25.0±2.7%, p<0.05) (Fig 2B and 2C). Following the decrease, 10 week Cyr61KD mean muscle content was only 0.5x MSC (25.0±2.7% vs. 47.2±0.6%). Cyr61KD graft muscle content was significantly lower than Cyr61OX at both 4 weeks (36.1±1.6% vs. 42.3±1.3%, p<0.05) and 10 weeks (25.0±2.7% vs. 40.2±2.4%, p<0.01). The density of the muscle and collagen fibers was slightly greater than control native tissue with density scores of 1.10±0.07 and 1.15±0.03 at 4 and 10 weeks for Cyr61KD and 1.11±0.02 and 1.16±0.03 for Cyr61OX (control native tissue = 1).

Wnt5aOX resulted in ~50% muscle content earlier than MSC (51.5±1.7% vs. 38.4±1.0% at 4 weeks, 1.3x MSC levels). A 4 to 10 week decrease led to mean muscle content of 45.7±1.7% at 10 weeks, comparable to MSC (47.2±0.6%). The Wnt5aOX pattern of early increased muscle content, followed by a decrease but maintenance of >45% mean muscle content, is similar to that seen previously with the MSC/CD34^+^ HSPC construct (55.3±0.9% at 4W; 50.1±1.9% at 10W) [7]. For Wnt5aOX, a density score of 1.27±0.07 at 4 weeks and 1.19±0.06 at 10 weeks confirmed the observation that, although the muscle percentage was similar to control native tissue (52.2±1.0%), muscle and collagen were more densely packed than control native tissue (control density score = 1).

### Peripheral Nerve Characterization

**Fig 3:**
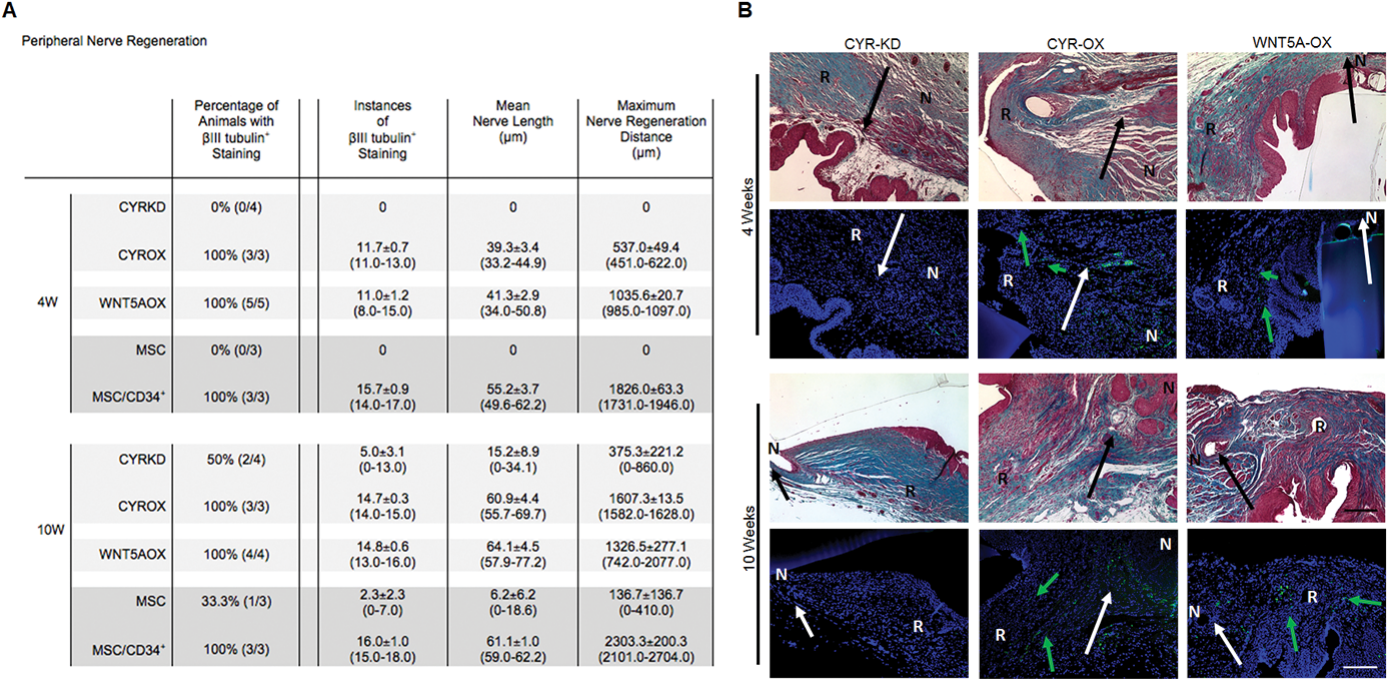
Effects of Cyr61 and Wnt5a on peripheral nerve regeneration. (A) Cyr61KD and MSC demonstrate no nerve regeneration at 4 weeks and poor nerve regeneration at 10 weeks. Wnt5aOX and MSC/CD34^+^ HSPC demonstrate increased early and robust nerve regeneration. Cyr61OX demonstrates nerve regeneration that continues to improve from 4 to 10 weeks. Data shown as means ± SEM (range). MSC and MSC/CD34^+^ HSPC data represent new quantitative assessment for a subset of samples from a previous study.[7] (B) Sample photomicrographs demonstrate βIII tubulin^(+)^ (green) neuronal staining (rows 2 and 4, blue: DAPI, green arrows: regenerated nerves, white arrows: transition between native and regenerated tissue, R: regenerated tissue, N: native tissue). Masson’s trichrome-stained images are of a serial section of tissue for each sample (rows 1 and 3; black arrows: transition between native and regenerated tissue). Scale bar, 200 μm. (Unseeded, MSC and MSC/CD34^+^ HSPC images shown in S2 Fig).

Previously, no evidence of peripheral nerve regeneration was detected in regenerated MSC graft tissue at 4 or 10 weeks [7]. However, combining MSCs with CD34^+^ HSPCs resulted in graft tissue with substantial peripheral nerve regeneration at 4 weeks [7]. To further characterize peripheral nerve regeneration in both current cell lines and a subset of previous cell lines, instances of βIII tubulin^+^ staining in regenerated tissue were counted and measured (length), and maximum nerve regeneration distance was determined. In this assessment, minimal staining was observed for a single animal from the 10 week MSC group (n = 3); no positive staining was detected in the remaining two 10 week animals or in any of the three 4 week animals (Fig 3, S2 Fig). Consistent with previous findings, instances of βIII tubulin^+^ staining representing peripheral nerves were found in all animals (n = 3 at each time-point) from the MSC/CD34^+^ HSPC group (15.7±0.9 at 4W; 16.0±1.0 at 10W).

Cyr61KD resulted in a very slight increase over MSCs, with no βIII tubulin^+^ staining at 4 weeks and two of four animals demonstrating minimal staining at the later time- point (Fig 3). Cyr61OX resulted in comparatively robust peripheral nerve regeneration (determined to be derived from native tissue), with instances of βIII tubulin^+^ staining in all animals at both time-points (11.7±0.7 at 4W; 14.7±0.3 at 10W). Regeneration was substantial but less rapid than MSC/CD34^+^ HSPCs, with lower mean nerve length and maximum nerve regeneration distance at 4 weeks (39.3±3.4μm vs 55.2±3.7μm; 537.0±49.4μm vs 1826.0±63.3μm), but similar mean nerve length and 0.7x maximum nerve regeneration distance by 10 weeks (60.9±4.4μm vs 61.1±1.0μm; 1607.3±13.5μm vs 2303.3±200.3μm).

Wnt5aOX also promoted peripheral nerve regeneration, with instances of βIII tubulin^+^ staining in all animals at both time-points (11.0±1.2 at 4W; 14.8±0.6 at 10W). A strong early response was observed although the degree of nerve regeneration did not quite reach MSC/CD34^+^ HSPC levels at 4 weeks (mean nerve length 41.3±2.9μm vs 55.2±3.7μm; mean maximum nerve regeneration distance 1035.6±20.7μm vs 1826.0±63.3μm). By 10 weeks, mean nerve length was comparable to MSC/CD34+ HSPCs (64.1±4.5μm vs 61.1±1.0μm), but maximum nerve regeneration distance remained reduced (1326.5±277.1μm vs 2303.3±200.3μm).

### Urothelium Quantification

**Fig 4:**
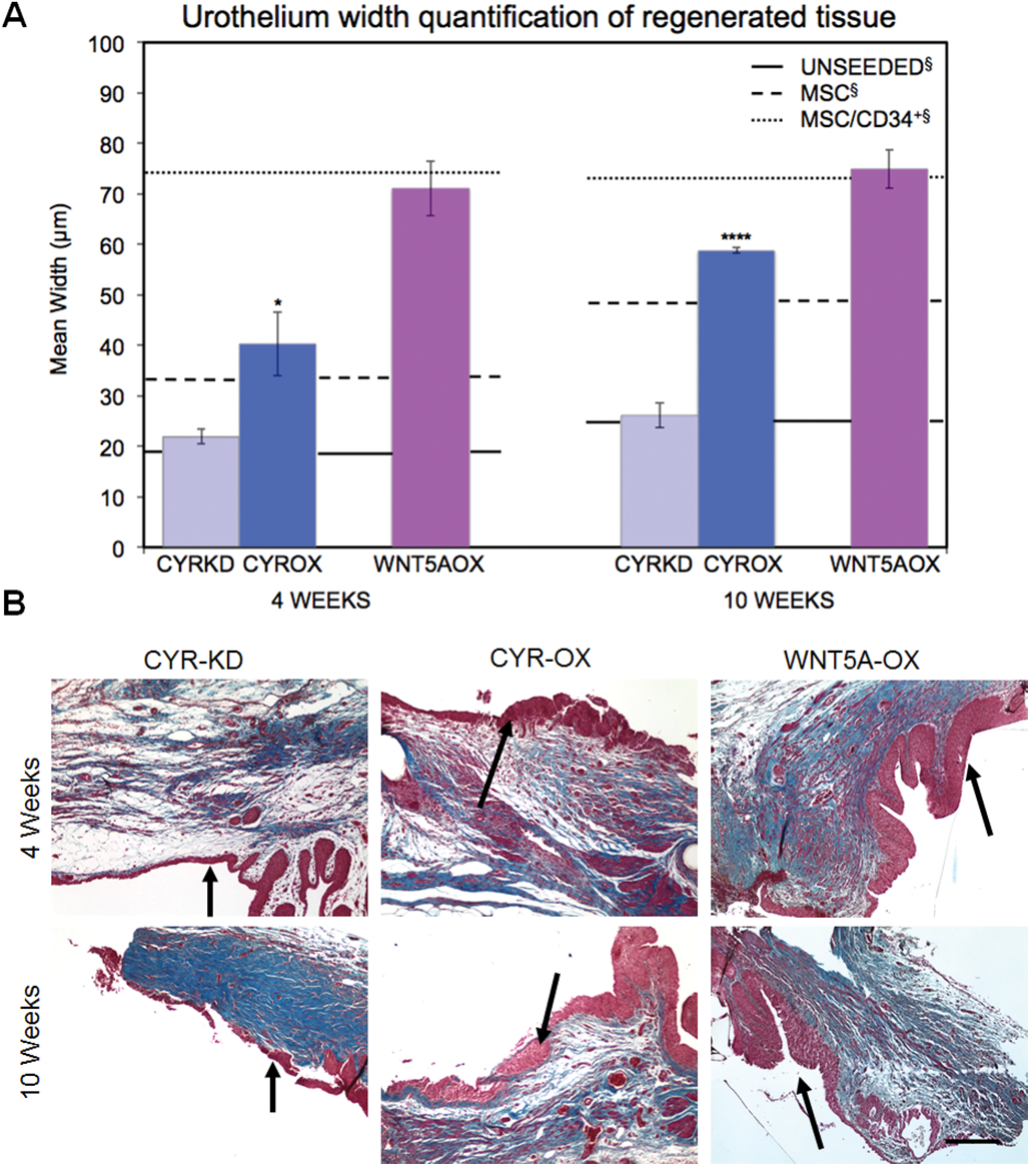
Effects of Cyr61 and Wnt5a on urothelium growth. (A) Wnt5aOX resulted in mean urothelium width similar to MSC/CD34^+^ HSPCs. Cyr61OX resulted in urothelium width significantly greater than Cyr61KD at both time points. Cyr61KD and unseeded grafts had the thinnest urothelium. Data shown as means ± SEM; *P<0.05 and ****P<0.0001 for Cyr61KD vs Cyr61OX. Unseeded, MSC and MSC/CD34^+^ HSPC data represent new quantitative assessment for a subset of samples from a previous study.[7] (B) Sample photomicrographs demonstrate thickened urothelium with homogenously small nuclei consistent with urothelial hyperplasia in the Cyr61OX and Wnt5aOX groups. Black arrows mark the transition between regenerated and native tissue. Scale bar, 200 μm. (Unseeded, MSC and MSC/CD34^+^ HSPC images shown in S2 Fig).

Previous findings demonstrated that grafts seeded with both MSCs and CD34^+^ HSPCs displayed greater urothelium thickness than grafts with MSCs alone [7]. The urothelial layer from a subset of these earlier cell lines, along with urothelial tissue from the current cell lines, was examined further to allow quantification of thickness. As expected, mean urothelium width for MSC grafts was lower than for MSC/CD34^+^ HSPC grafts at both time-points (33.1±1.3μm vs 74.3±4.5μm, p<0.01 at 4W; 48.6±3.4μm vs 73.1±6.0μm, p<0.05 at 10W) (Fig 4). A significant increase in thickness from 4 to 10 weeks was observed only for MSC grafts (33.1±1.3μm vs 48.6±3.4μm, p<0.05). Cyr61OX resulted in urothelium width greater than, but within the range of, MSC at both time-points (40.3±6.3μm vs 33.1±1.3μm at 4W; 58.8±0.6μm vs 48.6±3.4μm at 10W). Cyr61KD resulted in urothelial thickness significantly less than Cyr61OX (21.9±1.5μm vs 40.3±6.3μm, p<0.05 at 4W; 26.1±2.4μm vs 58.8±0.6μm, p<0.0001 at 10W), and did not increase over time; accordingly, at 10 weeks Cyr61KD mean urothelial width was only 0.5x MSC (26.1±2.4μm vs 48.6±3.4μm, p<0.001). Cyr61KD urothelial thickness was similar to unseeded POC (21.9±1.5μm vs 19.5±3.3μm at 4W; 26.1±2.4μm vs 25.7±4.2μm at 10W) (S3 Fig). Wnt5aOX resulted in a pattern of urothelium regeneration similar to that observed in MSC/CD34^+^ HSPC grafts, with a strong early response but no additional thickening over time (71.0±5.4μm vs 74.3±4.5μm at 4W; 74.9±3.8μm vs 73.1±6.0μm at 10W).

### Urodynamics

**Fig 5:**
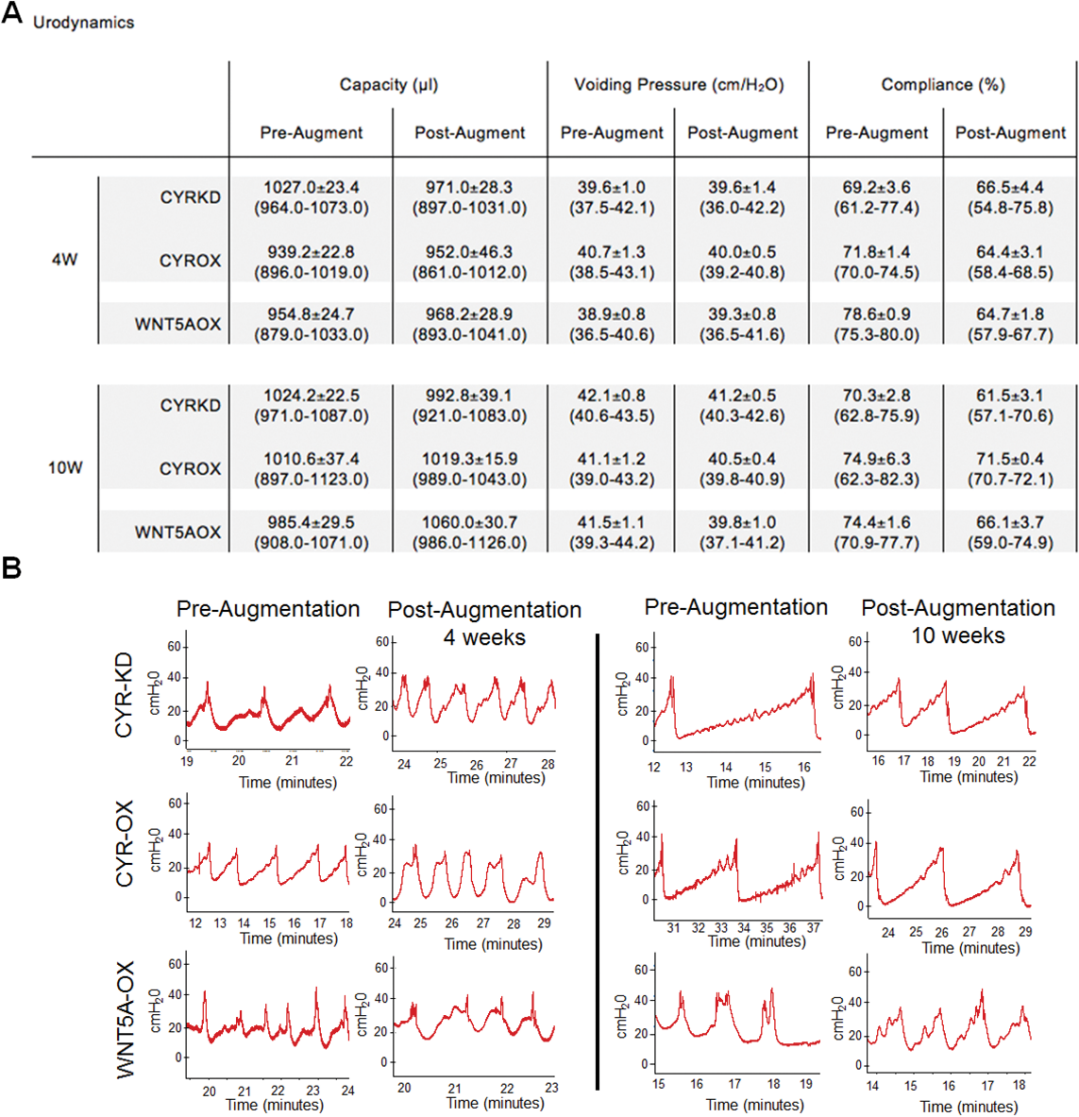
Effects of Cyr61 and Wnt5a on urodynamics. (A) Data shown as means ± SEM (range) for pre- and post-augment capacity (volume infused prior to first urine leak), voiding pressure (highest pressure at time of void) and compliance (percentage of bladder volume infused at pressures ≤ 20 cmH_2_O). By 4 weeks, Cyr61KD, Cyr61OX and Wnt5aOX groups all demonstrated >90% mean percent recovery of pre-augment bladder capacity [(post-augment capacity–pre-augment capacity)*100]. (B) Representative tracings from urodynamics evaluation. Voiding pressures averaged 40 cm H_2_O.

Bladder capacity, voiding pressure and compliance (the percentage of bladder filling at lower than 20 cm H_2_O) were measured pre- and post-augment. Voiding pressures were consistent across all time-points and groups. For all groups, mean percent recovery of bladder capacity was >90% by 4 weeks (percent recovery calculated as [(post-augment capacity–pre-augment capacity)*100]). A possible trend toward decreased compliance post-augment was observed (Fig 5).

## Discussion

Efforts to regenerate functional bladder tissue have been met by confounding results in both basic science and clinical arenas. Although many tissue engineering-based strategies have been employed to attain this goal, discouraging data from a recent clinical trial would suggest that alternative strategies must continue to be pursued to meet this great unmet clinical need.[6] A highly novel study recently performed by our group utilized spina bifida derived bone marrow MSCs in combination with donor-matched CD34^+^ HSPCs in an attempt to regenerate bladder tissue in vivo.[7] Although the results from this study demonstrated the unique positive synergistic effects of combining both cell populations with regards to bladder tissue regeneration, the study mainly evaluated the regeneration of bladder tissue at the macro- and microscopic levels. Thus, in-depth analyses regarding the elucidation of molecular pathways crucial to bladder tissue regeneration were not examined. Within the context of this study, the pro-angiogenic Cyr61 gene and the pleiotropic Wnt5a gene demonstrated profound effects on multiple aspects of bladder tissue regeneration in our in vivo model. Specifically, as compared to Cyr61OX grafts, regenerating bladder tissue from Cyr61KD constructs demonstrated greater collagen accumulation, diminished vasculature, the reduced ability to regenerate peripheral nerves, and decreased urothelium width. Conversely, as compared to Cyr61KD grafts, Cyr61OX constructs induced increased bladder smooth muscle and urothelium regeneration as well as peripheral nerve regeneration. Nonetheless, neither Cyr61OX nor Cyr61KD constructs achieved levels of tissue regeneration demonstrated by MSC/CD34^+^ HSPC seeded constructs with the exception of Cyr61OX-stimulated vascular growth at the later time-point. Contrastingly, Wnt5aOX constructs performed similarly than MSC/CD34^+^ HSPC seeded constructs across measures suggesting that the varied role of Wnt5a affects multiple aspects of bladder regeneration. Urodynamic testing of augmented bladders did not show significant differences between Cyr61KD, Cyr61OX and Wnt5aOX constructs in several bladder functional parameters including voiding pressure and capacity. The complicated results observed with the Cyr61 studies shifts our focus upon the examination of downstream molecular players affected by Wnt5a signaling in order to gain a better understanding of the molecular landscape involved in bladder tissue regeneration.

Functional neo-vascularization of primitive tissues undergoing regeneration is a major obstacle for contemporary tissue regeneration strategies.[7, 46] Cells localized at the center of large grafts are more likely to experience ischemic cell death given the limitations of oxygen and nutrient diffusion resulting in subsequent graft failure. The blood vessel growth-promoting features of Cyr61 have been shown to be essential in a variety of in vivo models including a rabbit based model of musculoskeletal regeneration.[50] Frey et al describe the positive effects of Cyr61 coated scaffolds used to envelope a simulated osteotomy of the rabbit femur. Treated animals underwent statistically significant increases in callus diameter and increased torsional strength as compared to controls. The evidently uncomplicated cause and effect relationship of Cyr61 application seen in the Frey study was not as readily straightforward during the present study. Cyr61 appeared to play a very complex role in various aspects of bladder tissue regeneration as exemplified by vasculature and muscle quantification data. Tissue vascularization data suggests there was an approximate 2.5 fold reduction in vessel number when comparing Cyr61KD constructs to Cyr61OX constructs. However, Cyr61KD constructs still maintained a level of vasculature at both time-points that was able to sustain regenerating bladder tissue. This may have been attributed to several scenarios including the limited stoichiometric levels of Cyr61 being produced by these constructs that still met a minimum threshold level to initiate angiogenesis and maintain post-angiogenic events. It is also plausible that secondary compensatory angiogenic mechanisms went into effect, albeit in a mild manner, again to attain minimum levels to promote vessel growth. Multiple redundant mechanisms have been identified that could initiate angiogenesis and maintain vascular–related events post-angiogenesis.[51, 52] These include VEGF independent and FGF mediated pathways that are evident in solid tumor development under hypoxic conditions that demonstrate unregulated vessel growth.[51, 53] The seemingly mild effects that Cyr61KD constructs had on vasculature growth were antithetical with regards to bladder muscle and urothelium development. At the terminal time-point of the study, Cyr61KD constructs contained approximately 75% collagen which is in complete contrast to normal bladder muscle to collagen ratios that approach 1:1 in a variety of species.[54] This was accompanied by a morphologically thinner and frail urothelial layer with a mean width of approximately 25μm. This data approaches levels found in unseeded POC scaffolds in which poor bladder tissue regeneration was observed.[45] The limited levels of blood vessel regeneration also affected peripheral nerve regeneration in deleterious manner. As there was no evidence of peripheral nerve regeneration at the initial time-point, scant levels of peripheral nerve tissue became apparent by 10 weeks but had no statistical difference compared to unmanipulated MSC constructs. As blood vessels are typically located within close proximity to nerves in order to serve as a nutrient source, [55, 56] it is not surprising that there was a poor peripheral nerve regenerative response within these constructs.

Cyr61OX constructs, on the other hand, exceeded vasculature and peripheral nerve regeneration parameters demonstrated in previous MSC-seeded groups and even surpassed vasculature levels at 10 weeks that were similar to peak 4 week levels attained by MSC/CD34^+^ HSPC constructs. Despite this, the over-expression of Cyr61 resulted in unorganized, highly tortuous vessels similar to those seen in developing solid tumors that were leaky in nature and led to the formation of hematomas throughout the bladder.[57, 58] The lack of structural integrity of the blood vessels may have been caused by the dissolution of the tight junctions between cells mediated by Ang1 and Syx, for example, thus negatively affecting the proper function of the vessels.[59] Surprisingly, our observations regarding vascularity and peripheral nerve regeneration did not coincide with statistically relevant functional gains as measured by urodynamics. The lack of functional differences may result from the small size of the graft, where even sub-optimal angiogenesis could be adequate to prevent central graft ischemia and/or that the remaining native bladder tissue was resilient enough to maintain normal bladder function. Cyr61 data taken as a whole would suggest that more studies are required to decipher its role in bladder tissue regeneration.

Wnt5a signaling independent of β-catenin involvement plays numerous roles ranging from organ system genesis and development to the modulation of cell planar polarity to aspects of stem cell regulation throughout the lifespan of many organisms.[60–63] Over-expressing Wnt5a constructs utilized in our studies were able to reconstitute several pivotal bladder tissue components including vasculature, smooth muscle content, urothelium and peripheral nerves in bladder tissue undergoing regeneration. Quantitative data exemplifying the regenerative potential of Wnt5aOX constructs with regards to aforementioned tissues rivaled those that were previously described in MSC/CD34^+^ HSPC seeded constructs used for augmentation. Regenerated blood vessels were patent, appeared to sustain blood flow as demonstrated by capillaroscopy, and could be grossly described as a widely distributed meshwork of blood vessels encompassing regenerated bladder tissue. There was no evidence of blood vessel leakiness as experienced with Cyr61OX constructs while the tortuosity of the vessels, which is indicative of angiogenesis, was observable to a lesser degree than those found in Cyr61OX constructs. Intriguingly, our previous studies support the notion that Wnt5a plays a role in the angiogenic response within our bladder regeneration model. The MSC/CD34^+^ HSPC seeded grafts express Wnt5a in the proximity of blood vessels undergoing angiogenesis, with concurrent expression of its receptor, Fzd4, on the vasculature itself.[29] As Wnt proteins have been shown to function in a paracrine fashion in a dose-dependent manner, it may be plausible to surmise that a concentration gradient of Wnt5a could have been established during this regenerative process providing stimulus for vessels to grow and develop. Unfortunately, due to the inability to create a functional Wnt5aKD construct, a true head-to-head comparison against Wnt5aOX constructs could not be achieved. The failure of Wnt5aKD MSCs to thrive in vitro is hardly surprising as other studies have shown the requirement of Wnt5a ranging from the cellular to organismal levels. This is clearly demonstrated by the absence of Wnt5a expression which is perinatal lethal in homozygous null knockout mice while heterozygous counterparts suffer from severe malformations at the gross and microscopic levels in heterozygous counterparts. [64, 65]

The interior lumen of the bladder is lined with urothelium, a unique tissue which protects the bladder from urine and multiple urogenic pathogens.[66, 67] We observed that Wnt5aOX constructs demonstrated the greatest urothelial thickness which was comparable to that observed in the previously described composite MSC/CD34^+^ HSPC grafts. This could have implications in the clinical setting where larger grafts may require more robust urothelium regeneration both in order to protect the regenerating tissue at the geographical center of the grafts and to minimize the risk of urine leaking into the abdominal cavity, a significant source of morbidity seen in a recent clinical trial of autologous bladder cells seeded onto a scaffold and used to augment native bladder tissue.[6] Normal human urothelium is 2–4 cells thick if stretched and 4–7 if contracted. In clinical practice, thickened urothelial lesions can be non-malignant as is the case with urothelial hyperplasia where the urothelium is thickened due to an increased number of normal cells with normal organization and homogeneous small nuclei. In contrast, urothelial carcinoma is characterized by a thickened urothelium exhibiting disorganized cells, large hyperchromatic nuclei and multiple mitotic figures. In all groups, we observed normal sized urothelial cells with accompanying nuclei and normal organization leading us to believe that the thickened urothelium seen in the Cyr61OX, Wnt5aOX, MSC, and MSC/CD34^+^ HSPC groups was secondary to urothelial hyperplasia as compared to hypertrophy or carcinoma in situ. While thicker urothelium has no clinical advantage in a normal bladder, a more robust urothelial regenerative response is likely to be clinically important in regenerative medicine where the normal urothelial regrowth is unable to reach the center of the grafts. The ability to improve urothelial coverage over the entire graft is vital given that this barrier reduces the likelihood of a urine leak. Urine penetrating the graft is not only toxic for seeded cells but causes significant clinical morbidity.[6]

Interestingly, our findings are in contrast with studies performed by Mysorekar et al with regards to Wnt5a signaling in urothelial regeneration. Data from their study demonstrate that the highly restricted expression of endogenous Wnt5a initiates urothelium regeneration.[68] Following the infection of mice with FimH^+^ uropathogenic E. coli (UPEC), the virulent bacteria attached to the urothelium via FimH adhesin and triggered the rapid differentiation of basal and intermediate urothelial cells into terminally differentiated umbrella cells. These events occurred in concert with the diminished expression of the Ca^2+^ dependent Wnt5a signaling cascade suggesting Wnt5a may be a checkpoint gene with regards to urothelial differentiation. This may be attributed to several factors including the mechanism in which regeneration was initiated and subsequently achieved. In the Mysorekar study, FimH^+^ UPEC was the insulting factor that provided the stimulus for regeneration following cell exfoliation while our study utilized a specifically created surgical defect combined with genetically modified cells. The different modes of urothelial injury and regeneration should be further investigated as this could have significant clinical impact in the bladder replacement or augmentation setting. Secondly, the roles of other Wnt genes have also been implicated in urothelium regeneration. Shin et al describe the role of the Wnt/Shh signaling pathway in an UPEC induced bladder injury model.[69, 70] However, those studies implicated the roles of Wnt2 and Wnt4 suggesting their ability to influence urothelial regeneration as mediated through the adjacent stroma. Interestingly, Wnt2 serves as the ligand for Fzd4 receptor similar to Wnt5a and may function in an analogous manner in this situation.[71, 72] To our knowledge, this is the first study that describes the pleiotropic effects of Wnt5a and its direct relationship to multiple aspects of bladder tissue regeneration that include blood vessels, peripheral nerve, smooth muscle and urothelium in a bladder augmentation model.

Our findings suggest that MSC-based bladder tissue regeneration is impacted by the modified expression of Cyr61 and Wnt5a. However, the complicated results attained with Cyr61 constructs pose a dilemma as to whether the application of this protein would actually be relevant in a clinical setting. Furthermore, modulating the concentration and/or timing of Wnt5a expression will be important for optimizing tissue regeneration. As the previous use of a simplistic biodegradable scaffolds combined with a pathological cell source proved ineffective in clinical trials, future strategies for tissue-engineered bladder need to be re-evaluated with regards to the underlying cell sources along with scaffold design and implementation. Finally, the number of cells and cell types used should also be taken into consideration when making comparisons between MSC, MSC/CD34+ HSPC, and the genetically modified cell groups. The addition of the CD34+ HSPC population to the constructs provides not just a source of cytokine, namely Wnt5a, but a battery of other factors that could affect regeneration. The goal of this study was to determine the role of Wnt5a and its effects on bladder regeneration. Altering the number of CD34+ HSPCs may provide a different landscape in the regenerative process and is the subject of future studies.

## Conclusion

Within this study, we have demonstrated that both Cyr61 and Wnt5a are potent extracellular signaling molecules whose expression directly influences several salient features of bladder tissue regeneration. Coerced expression of Wnt5a in grafted MSCs closely mimicked the previously encouraging findings observed with MSC/CD34^+^ HSPC co-transplantation. The elucidation of the role of Wnt5a during this regenerative process may represent a putative mechanism by which implanted CD34^+^ HSPCs interact with MSCs to improve multiple outcome parameters. This further suggests that Wnt5a may act in lieu of CD34^+^ HSPCs and opens up the realm of possibilities with regards to the in vivo delivery of Wnt5a, be it by functionalized scaffolds or nanoparticle systems.

## Supporting Information

S1 Fig Representative photomicrographs of regenerated vasculature and musculature of unseeded, MSC (unmanipulated) and MSC/CD34^+^ HSPC grafts (similar to previously reported [7]). Photomicrographs demonstrate that at 4 weeks MSC/CD34^+^ HSPC grafts had mean muscle content 1.4x MSC grafts and 2.9x unseeded grafts, with a greater number of vessels/mm^2^ and higher percent vasculature (data previously reported [7]). Scale bar, 50 μm.
(TIF)

S2 Fig Representative photomicrographs of regenerated peripheral nerves of unseeded, MSC (unmanipulated) and MSC/CD34^+^ HSPC grafts (similar to previously reported [7]). Unseeded grafts at 4 and 10 weeks and MSC grafts at 4 weeks had no identified peripheral nerve regeneration. MSC/CD34^+^ HSPC grafts demonstrated increased early and robust nerve regeneration with βIII tubulin^(+)^ (green) neuronal staining (rows 2 and 4, blue: DAPI, green arrows: regenerated nerves, white arrows: transition between native and regenerated tissue, R: regenerated tissue, N: native tissue). Masson’s trichrome-stained images are of a serial section of tissue for each sample (rows 1 and 3; black arrows: transition between native and regenerated tissue). Scale bar, 200 μm.
(GIF)

S3 Fig Representative photomicrographs demonstrating urothelium regrowth of unseeded, MSC (unmanipulated) and MSC/CD34^+^ HSPC grafts (similar to previously reported [7]). Photomicrographs demonstrate unseeded grafts with significantly thinner urothelium overlying regenerated tissue. Black arrows mark the transition between regenerated and native tissue. Scale bar, 200 μm.
(GIF)

S1 File Capillaroscopy of in vivo bladder tissue demonstrating blood flow through blood vessels that have undergone angiogenesis. (MP4)
