# Supplementary figures and images for: The Role of Genetically Modified Mesenchymal Stem Cells in Urinary Bladder Regeneration
Source: PLoS One. 2015 Sep 23;10(9):e0138643. doi: 10.1371/journal.pone.0138643 (PMC4580420; doi:10.1371/journal.pone.0138643)

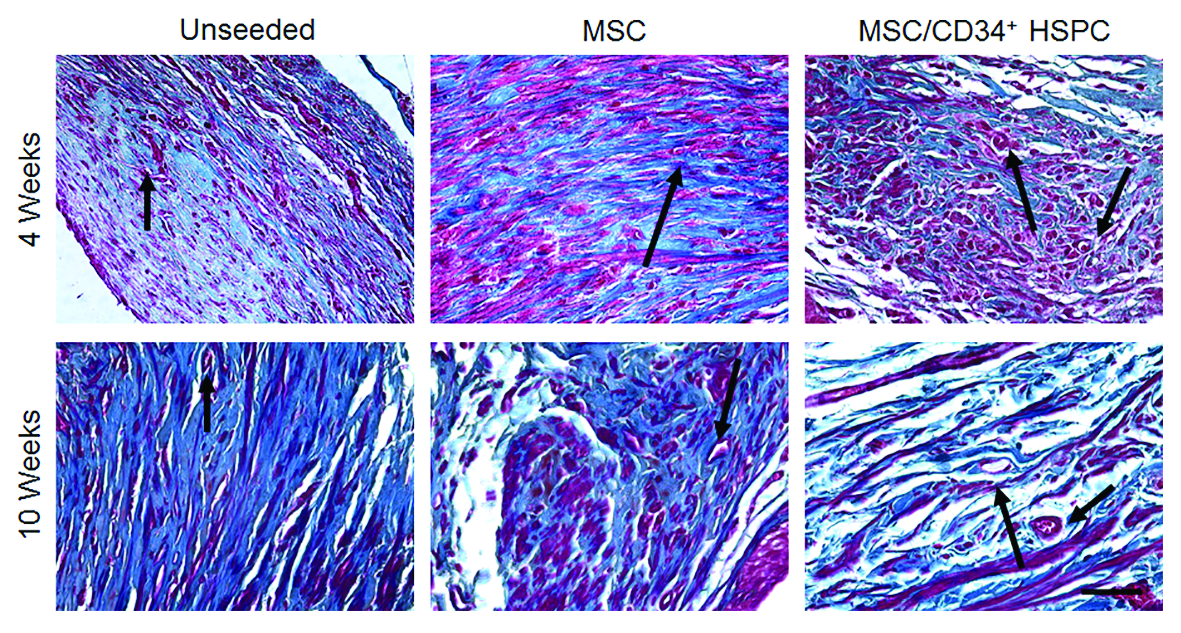

Supplement: S1 Fig — Photomicrographs demonstrate that at 4 weeks MSC/CD34+ HSPC grafts had mean muscle content 1.4x MSC grafts and 2.9x unseeded grafts, with a greater number of vessels/mm2 and higher percent vasculature (data previously reported [7]). Scale bar, 50 μm. (TIF) [file pone.0138643.s001.tif]

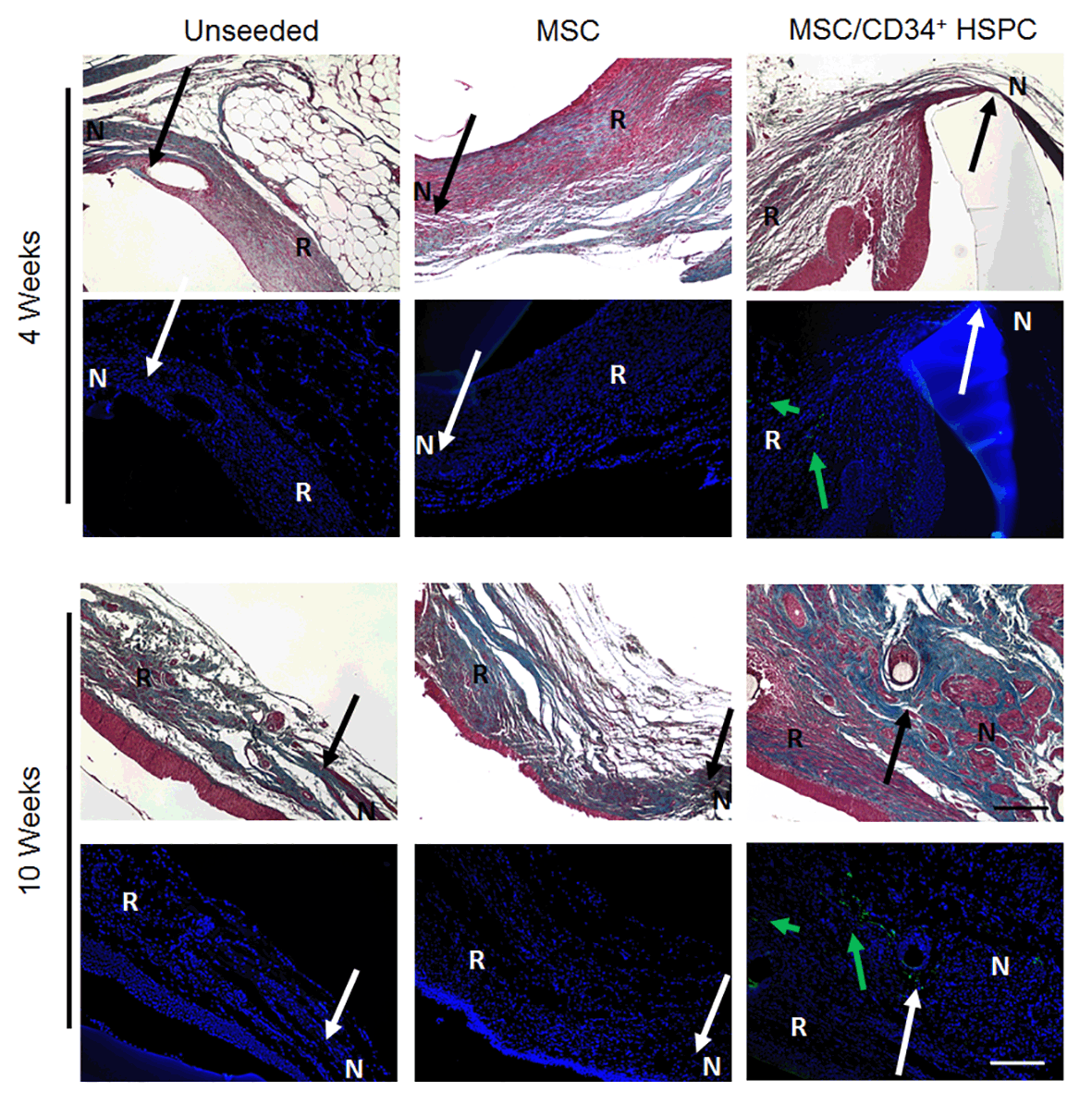

Supplement: S2 Fig — Unseeded grafts at 4 and 10 weeks and MSC grafts at 4 weeks had no identified peripheral nerve regeneration. MSC/CD34+ HSPC grafts demonstrated increased early and robust nerve regeneration with βIII tubulin(+) (green) neuronal staining (rows 2 and 4, blue: DAPI, green arrows: regenerated nerves, white arrows: transition between native and regenerated tissue, R: regenerated tissue, N: native tissue). Masson’s trichrome-stained images are of a serial section of tissue for each sample (rows 1 and 3; black arrows: transition between native and regenerated tissue). Scale bar, 200 μm. (GIF) [file pone.0138643.s002.gif]

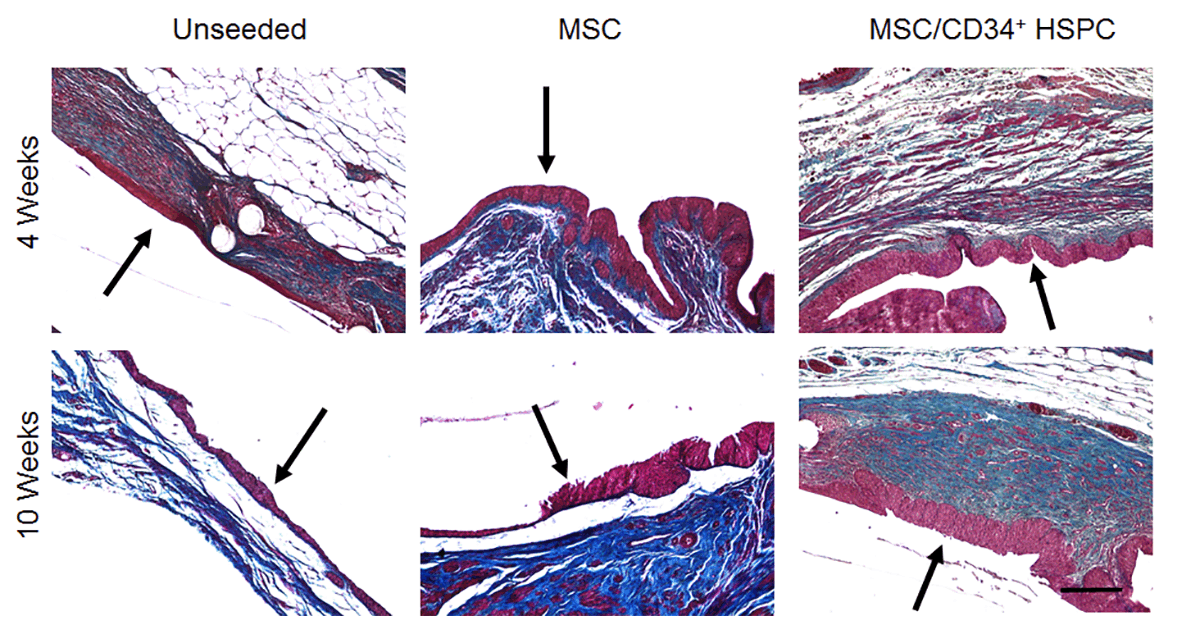

Supplement: S3 Fig — Photomicrographs demonstrate unseeded grafts with significantly thinner urothelium overlying regenerated tissue. Black arrows mark the transition between regenerated and native tissue. Scale bar, 200 μm. (GIF) [file pone.0138643.s003.gif]
